# Accumulation of advanced oxidative protein products exacerbate satellite glial cells activation and neuropathic pain

**DOI:** 10.1186/s10020-025-01076-x

**Published:** 2025-01-26

**Authors:** Chen Tu, Shi-Cheng Wang, Meng-Xuan Dai, Si-Qi Lai, Zhi-Wei Huang, Yong-Peng Yu, Yun-Biao Chen, Ji-Huan Zeng, Liang Wang, Zhao-Ming Zhong

**Affiliations:** 1 Department of Spine, Center for Orthopedic Surgery, The Third Affiliated Hospital of Southern Medical University, Guangzhou, China; 2 Division of Spine Surgery, Department of Orthopedics, Nanfang Hospital, Southern Medical University, 1838 North Guangzhou Ave, Guangzhou, 510515 People’s Republic of China; 3 Department of Pathology, Center for Orthopedic Surgery, The Third Affiliated Hospital of Southern Medical University, Guangzhou, China; 4 Jiangxi Provincial People’s Hospital, The First Affiliated Hospital of Nanchang Medical College, Nanchang, China

**Keywords:** AOPPs, SGCs, RAGE, Inflammation, Neuropathic pain

## Abstract

**Background:**

Neuropathic pain (NP) is a debilitating condition caused by lesion or dysfunction in the somatosensory nervous system. Accumulation of advanced oxidation protein products (AOPPs) is implicated in mechanical hyperalgesia. However, the effects of AOPPs on NP remain unclear.

**Methods:**

A rat model of NP was established by chronic constriction injury (CCI) and employed to evaluate the changes of mechanical withdrawal threshold, thermal and cold withdrawal latency, as well as AOPPs levels. The effects of AOPPs on the activation of satellite glial cells (SGCs) in the dorsal root ganglion (DRG), receptor for advanced glycation end-products (RAGE) expression, and NF-κB signaling pathway activation were also investigated using western blotting, immunofluorescence, and the Fluo4-AM fluorescence probe for calcium signaling. Additionally, oxidative stress levels and inflammatory cytokine production in SGCs, triggered by AOPPs exposure, were measured through the DCFH-DA probe for ROS detection and ELISA kits for cytokine quantification.

**Results:**

CCI significantly elevated the AOPPs levels in the plasma and sciatic nerve and caused AOPPs accumulation in the DRG. Exogenous AOPPs activated SGCs, increased reactive oxygen species and inflammatory response, upregulated the RAGE, and activated NF-κB signaling. The RAGE inhibitor FPS-ZM1 effectively inhibited AOPPs-induced SGC activation. Additionally, AOPPs intervention worsened CCI-induced hyperalgesia and neuroinflammation in vivo.

**Conclusion:**

These results indicate that AOPPs exacerbate the SGC activation and NP following nerve injury, and AOPPs accumulation might play an important role in the pathogenesis of NP.

## Introduction

Neuropathic pain (NP) is a challenging disease resulting from damage or dysfunction of peripheral nerves (Thouaye and Yalcin 2023). Somatosensory neurons of the dorsal root ganglia (DRG) play a pivotal role in the development and maintenance of pain (Xu et al. 2022). These neurons are closely enveloped by satellite glial cells (SGCs), the most abundant non-neuronal cells in DRG. SGCs are interconnected via gap junctions composed of connexin 43 (Cx43) (Qiu et al. 2024). This connectional arrangement facilitates communication among SGCs and neighboring neurons (Retamal et al. 2017). When peripheral nerves are damaged, SGCs are activated, characterized by an increased expression of glial fibrillary acidic protein (GFAP) (Chen et al. 2022a). Activated SGCs enhance cell communication via Cx43 and secrete inflammatory mediators such as interleukin 1β (IL-1β) and tumor necrosis factor α (TNFα), increasing the excitability of somatosensory neuron cells and promoting the onset and maintenance of pain (Qiu et al. 2024; Zhao et al. 2023). The activation of SGCs, as part of the peripheral nervous system injury response, has been shown to be one of pathogenesis of NP (Tonello et al. 2023).

Nuclear factor κB (NF-κB), an inducible transcription factor, is recognized as a classical pro-inflammatory signaling pathway (DiDonato et al. 2012). NF-κB is composed of NF-κB1(p50) or NF-κB2(p52) bound to either RELA(p65), RELB, c-REL, the NF-κB1(p50)/ RELA(p65) heterodimer is the most abundant form (Liu et al. 2017). Under diverse stimuli, RELA(p65) is activated and regulates multiple target genes involved in cell proliferation, inflammatory cytokines, chemokines, and apoptotic mediators (Sun et al. 2013). In NP, NF-κB activation in microglial cell following sciatic nerve injury promotes the release of IL-1β and TNFα, contributing to the development of pain (Cheng et al. 2018). Recently, inhibiting NF-κB signaling in GFAP-high expressing SGCs has been shown to alleviate NP induced by nerve injury (Xie et al. 2021).

Advanced oxidation protein products (AOPPs) are dityrosine-containing protein cross-links formed during the oxidative modification of plasma proteins, including albumin, fibrinogen, and lipoproteins. They serve as widely recognized markers of oxidative stress in various diseases (Camilla et al. 2011; Czubilinska-Lada et al. 2022; Rasool et al. 2019). AOPPs accumulation also induces reactive oxygen species (ROS) generation (Li et al. 2023; Srinivas et al. 2019). Excessive ROS promote the progression of neuroinflammation and activate various ion channels in sensory neurons, such as TRPV1 and CGRP, inducing pain (Liu et al. 2024). The receptor for advanced glycation end products (RAGE) is a biological receptor for AOPPs, contributing to various diseases (Cao et al. 2014; Guo et al. 2008). Recent studies have demonstrated that RAGE expression in the DRG is implicated in nociception and NP (Araldi et al. 2024). Our previous study showed that AOPPs accumulation induced mechanical hyperalgesia in rats (Ding et al. 2016). However, the effects of AOPPs on the pathogenesis of NP warrant further investigation.

A chronic constriction injury (CCI) model of the rat sciatic nerve is known to exhibit NP behavior (Xia et al. 2021). Here, we established CCI rats to investigate the correlation between AOPPs accumulation and NP development. Next, we further explore the underlying mechanism by which AOPPs modulate NP.

## Materials and methods

### Animal experiments

Adult male Sprague–Dawley (SD) rats (3 months old) were purchased from the Animal Center of Southern Medical University. All animals were kept at room temperature (22 ± 2°C) under a 12-h light/dark cycle. Rats had ad libitum access to food and water (3 animals in each cage). After one week of acclimatization, the animals were randomly divided into two groups: CCI group or Sham group. Briefly, the rats were anesthetized with isoflurane, and the right sciatic nerve was exposed and loosely ligated three times with 4-0 silk sutures at 3 mm intervals, with each suture tied until a slight twitch in the bilateral hind limb was observed. In the sham group, the sciatic nerve was exposed but not ligated. Starting on the third day post-surgery, AOPPs (50 mg/kg/day) were administered intraperitoneally to the rats for four weeks. All experiments were approved by the Experimental Animal Care and Use Committee of Nanfang Hospital, Southern Medical University, and adhered to the guidelines of the International Association for the Study of Pain (Zimmermann 1983). The investigators were blinded to the group assignments for all assessments.

### AOPPs preparation

AOPPs were prepared following the methods described in previous studies (Tu et al. 2022). Briefly, a rat serum albumin (RSA) solution (20 mg/ml, Sigma, USA) was mixed with an equal volume of hypochlorous acid solution (40 mM, Fluka, Switzerland) and incubated in the dark at room temperature for 30 min. The prepared samples were then transferred to dialysis bags and dialyzed against phosphate-buffered saline (PBS, pH 7.4) to remove free hypochlorous acid. To eliminate contaminating endotoxins, all samples were passed through a detoxification chromatography column (Pierce, Rockford, IL).

### AOPPs detection

AOPPs were quantified as described previously (Tu et al. 2022). Briefly, 200 μl of the sample or chloramine-T was added to a 96-well plate, followed by the addition of 10 μl KI and 20 μl acetic acid. The absorbance at 340 nm was immediately measured using a microplate reader (Molecular Devices, USA). The concentration of AOPPs was expressed as chloramine-T equivalents in μM (plasma) or nmol/mg protein (sciatic nerve).

### Assessment of behavior

To assess mechanical hypersensitivity, a digital electronic von Frey aesthesiometer (IITC Life Science, CA, USA) was employed. Rats were initially acclimated to the environment in a transparent plastic chamber placed on a suspended metal grid for 15 min. Subsequently, the von Frey polypropylene tip was applied vertically to the midplantar surface of the hind paw, and the stimulus intensity was recorded. Positive responses, such as sudden withdrawal of the paw, licking, and shaking, were noted. The experiment was repeated five times, with 15-min intervals between each trial (Tu et al. 2024).

For the evaluation of thermal sensitivity, rats were placed on a temperature-controlled hot plate set at 45 °C. Response times for observed behavioral changes, such as paw licking, stamping, jumping, and escaping from the plate, were recorded. A cutoff time of 30 s was set. To prevent skin sensitization, the experiment was repeated five times, with each assessment separated by a 30-min interval (Tu et al. 2024).

### Primary satellite glial cells culture

SGCs were obtained and cultured as previously described (Tonello et al. 2023). In brief, DRG were harvested from 3-month-old rats and placed in cold DMEM/F12 medium. They were then digested with 0.25% trypsin and type I collagenase (Bio-sharp, China, 2 mg/ml) at 37 °C for 30 min. Following digestion, the DRG were dissociated using a 1 ml and then a 200 μl pipette tip, filtered through 40 µm and then 10 µm cell strainers, and cultured in DMEM/F12 medium containing 10% fetal bovine serum. After 24 h, the medium was replaced with fresh medium. Cultures were maintained at 37 °C with 5% carbon dioxide for approximately 10 days before use. To sustain the SGCs cultures, the DMEM/F12 medium was replaced every 2–3 days.

### Immunofluorescence staining

The lumbar (L2-5) DRG were fixed in 4% paraformaldehyde overnight at 4 °C. These DRG were then transferred to a 30% sucrose solution at 4 °C for 24 h. Subsequently, the DRG were embedded in optimal cutting temperature compound and sliced into 10 μm sections. To block nonspecific binding, the sections were treated with 5% goat serum at room temperature for 1 h. The sections were then incubated overnight at 4 °C with primary antibodies: rabbit anti-AOPPs (1:50, Cloud-clone, China), rabbit anti-Cx43 (1:400, Cell Signaling Technology, USA), rabbit anti-RAGE (1:500, Cell Signaling Technology, USA), and mouse anti-GFAP (1:400, Cell Signaling Technology, USA). Mouse/rabbit anti-glutamine synthetase (1:200, Abcam, UK) was used to label and identify SGCs, Mouse anti-Tubb3 (1:500, Abcam, UK) to label and identify DRG neuron. After primary antibody incubation, secondary antibodies conjugated with Alexa Fluor 488 (1:500, Thermo Fisher Scientific, USA) or Alexa Fluor 594 (1:500, Thermo Fisher Scientific, USA) were added and incubated at room temperature for 1 h. Finally, images were captured using a Nikon N31373 fluorescence microscope.

### Western blot

Cells were homogenized in RIPA buffer with 1 mM PMSF and protease inhibitors, and the supernatant was extracted by centrifugation at 4 °C and 12,000 rpm for 20 min. The protein concentration was determined using a bicinchoninic acid assay kit (Yeasen, China). Samples were separated by electrophoresis on a 10% sodium dodecyl sulfate–polyacrylamide gel and then transferred to polyvinylidene fluoride membranes (Millipore, Billerica, MA, USA). The membranes were blocked with 5% bovine serum albumin for 1 h at room temperature. Subsequently, the membranes were probed overnight at 4 °C with primary antibodies: rabbit anti-RAGE (1:800, Cell Signaling Technology, USA), rabbit anti-p65 (1:500, HUABIO, China), rabbit anti-phospho-p65 (1:800, HUABIO, China), rabbit anti-IκBα (1:400, HUABIO, China), and rabbit anti-GAPDH (1:5000, Abcam, UK). After washing, the membranes were incubated with the appropriate secondary antibody for 1 h, and protein bands were detected using chemiluminescence detection reagents (Yeasen, China).

### Quantitative real-time PCR

Total RNA was extracted using the EZ-press RNA Purification Kit (EZBioscience, USA) according to the manufacturer’s instructions. The RNA was reverse transcribed using the Color Reverse Transcription Kit with gDNA Remover (EZBioscience, USA). RT-qPCR analysis was performed on a LightCycler480 system using 2 × SYBR Green qPCR Mix (EZBioscience, USA). The following primer sequences were utilized: IL-1β: 5′-TTGAGTCTGCACAGTTCCCC-3′ (forward) and 5′-TCCTGGGGAAGGCATTAGGA-3′ (reverse), IL-6: 5′-CCCAACTTCCAATGCTCTCCT-3′ (forward) and 5′-GGATGGTCTTGGTCCTTAGCC-3′ (reverse), TNFα: 5′-GATCGGTCCCAACAAGGAGG-3′ (forward) and 5′-GCTTGGTGGTTTGCTACGAC-3′ (reverse), GAPDH: 5′-CCATCAACGACCCCTTCATT-3′ (forward) and 5′-CACGACATACTCAGCACCAGC-3′ (reverse).

### ROS detection

Intracellular ROS levels were detected using the fluorescent probe 2′,7′-dichlorofluorescein diacetate (DCFH-DA) (Biosharp, China) according to the manufacturer’s instructions (Tu et al. 2022). Briefly, the SGCs were incubated with 10μM DCFH-DA for 30 min in a dark incubator at 37 °C. Fluorescence intensity (Ex/Em = 488/525) was measured using a SpectraMax M5 system (Molecular Devices, USA) and visualized with confocal fluorescence microscopy (Olympus, Japan).

### Enzyme‑like immunosorbent assay (ELISA)

The rat IL-1β (PI303), IL-6 (PI328), and TNF-α (PT516) enzyme-linked immunosorbent assay (ELISA) kits were purchased from Beyotime (China). After collecting cell supernatant samples and centrifuging at 1000 rpm for 5 min, 100 μl of each sample was added to a 96-well plate. Detection reagents and wash solutions were added sequentially according to the assay kit instructions. Following incubation at room temperature in the dark for 30 min, the absorbance values of each well were measured using the SpectraMax M5 system (Molecular Devices, USA). A standard curve was prepared for each experiment.

### Calcium imaging

The calcium imaging followed the methodology outlined in a previous study (Qarot et al. 2024). SGCs were incubated with the Ca^2^⁺ indicator Fluo-4 AM (10 μM, excitation/emission spectra: 488/505, Yeeson, China) in DMEM/F12 medium, consistent with the culture conditions, for 30 min at 37 °C in an incubator. Following incubation, the cells were washed and allowed to de-esterify for an additional 30 min. Subsequently, the cells were mounted on a Zeiss LSM 980 laser scanning confocal microscope (Carl Zeiss, Germany), and baseline Fluo-4 AM fluorescence was recorded for 30 s. The medium was then replaced with an internal solution containing 2 µM free Ca^2^⁺, and the signal was recorded for 270 s. ImageJ software was utilized for image processing and analysis, including background signal subtraction and normalization of the Fluo-4 AM fluorescence signal to F/F0.

### Statistical analyses

Statistical analyses were performed using GraphPad Prism 9.30 (GraphPad Software, CA, USA). Differences between two groups were assessed using an unpaired Welch's t-test. For comparisons involving more than two groups, one-way analysis of variance (ANOVA) followed by the Bonferroni post hoc test was employed. All statistical tests were two-tailed, with significance set at *p* < 0.05. Normality of the data was evaluated using the Shapiro–Wilk or Kolmogorov–Smirnov tests. Data are presented as mean ± standard error of the mean (SEM).

## Results

### Accumulation of AOPPs is accompanied by persistent hyperalgesia in CCI rats

**Fig. 1:**
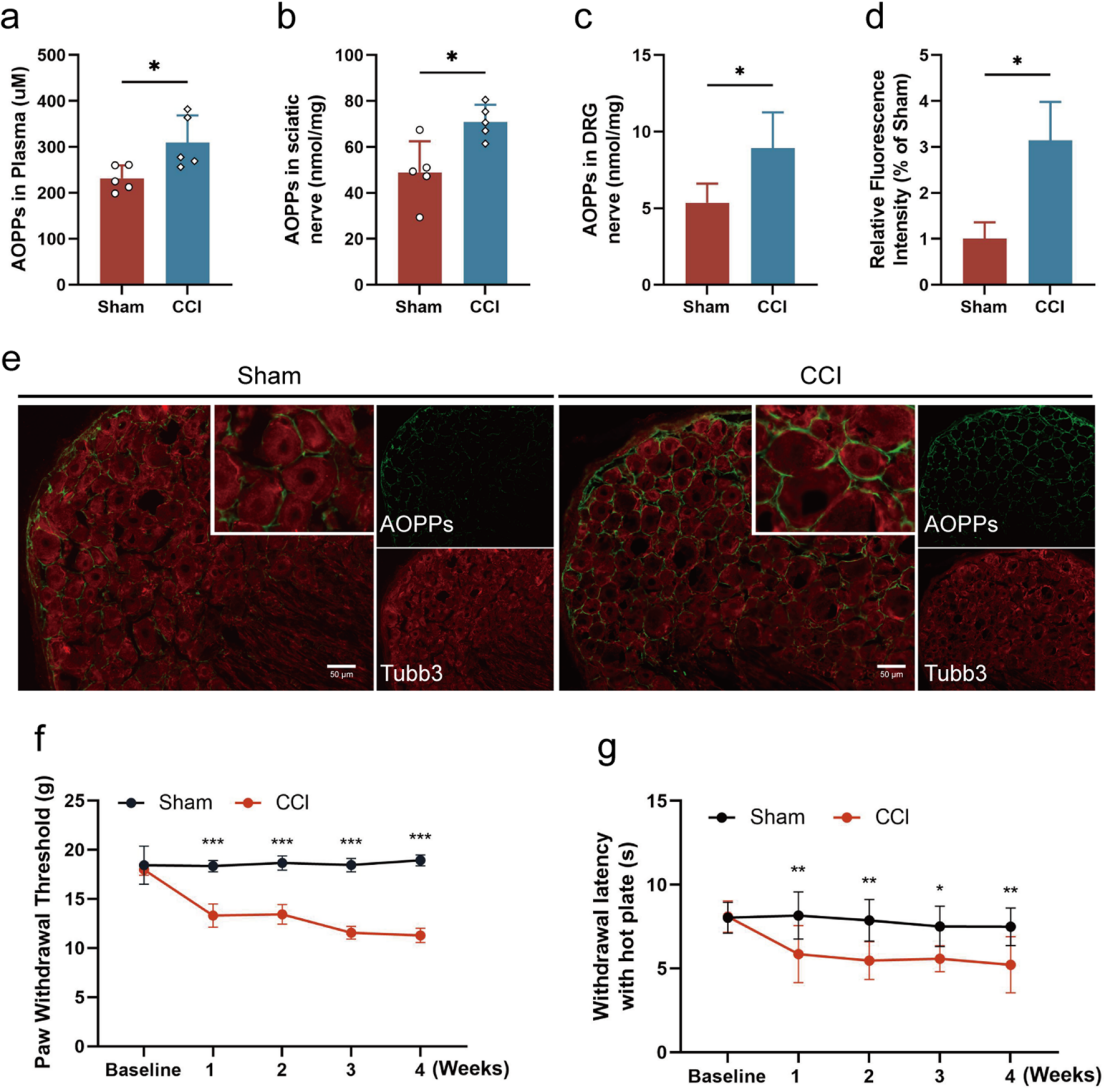
Accumulation of AOPPs is accompanied by persistent hyperalgesia in CCI rats. **a** Plasma AOPPs levels after 4 weeks of CCI and Sham surgery (n = 5, Welch’s *t*-test). **b** Sciatic nerve AOPPs levels following 4 weeks of CCI and Sham surgery (n = 5, Welch’s *t*-test). **c** DRG AOPPs levels following 4 weeks of CCI and Sham surgery (n = 5, Welch’s *t*-test). **d**, **e** Immunofluorescence showing the expression of AOPPs and Tubb3 in the L2–L5 DRG of CCI and Sham rats (scale bar, 50 μm), Fluorescence expression intensity of AOPPs was analyzed by ImageJ. **f** Mechanical withdrawal threshold (n = 8, two way-ANOVA), and **g** thermal withdrawal latency (n = 8, two way-ANOVA) following 4 weeks of CCI and Sham surgery. Data are presented as mean ± standard error of the mean (SEM). *p < 0.05, **p < 0.01, ***p < 0.001 vs Sham group. AOPPs, advanced oxidative protein products; CCI, chronic constriction injury; DRG, dorsal root ganglion; Tubb3, β-III tubulin

CCI, a common NP model, resulted in significantly elevated levels of AOPPs in the plasma (Fig. 1a), sciatic nerve (Fig. 1b) and DRG (Fig. 1c) of rats four weeks post-surgery compared to sham-operated rats. Immunofluorescence staining revealed that obvious accumulation of AOPPs was observed in the surrounding of DRG neurons in CCI rats compared to Sham rats (Fig. 1d, e). Concurrently, CCI rats exhibited persistent reductions in mechanical paw withdrawal thresholds (Fig. 1f) and thermal paw withdrawal latencies (Fig. 1g) compared to sham rats. These results suggest a close relationship between AOPPs levels and CCI-induced NP.

### SGCs activation and upregulated RAGE expression in the DRG of CCI rats

**Fig. 2:**
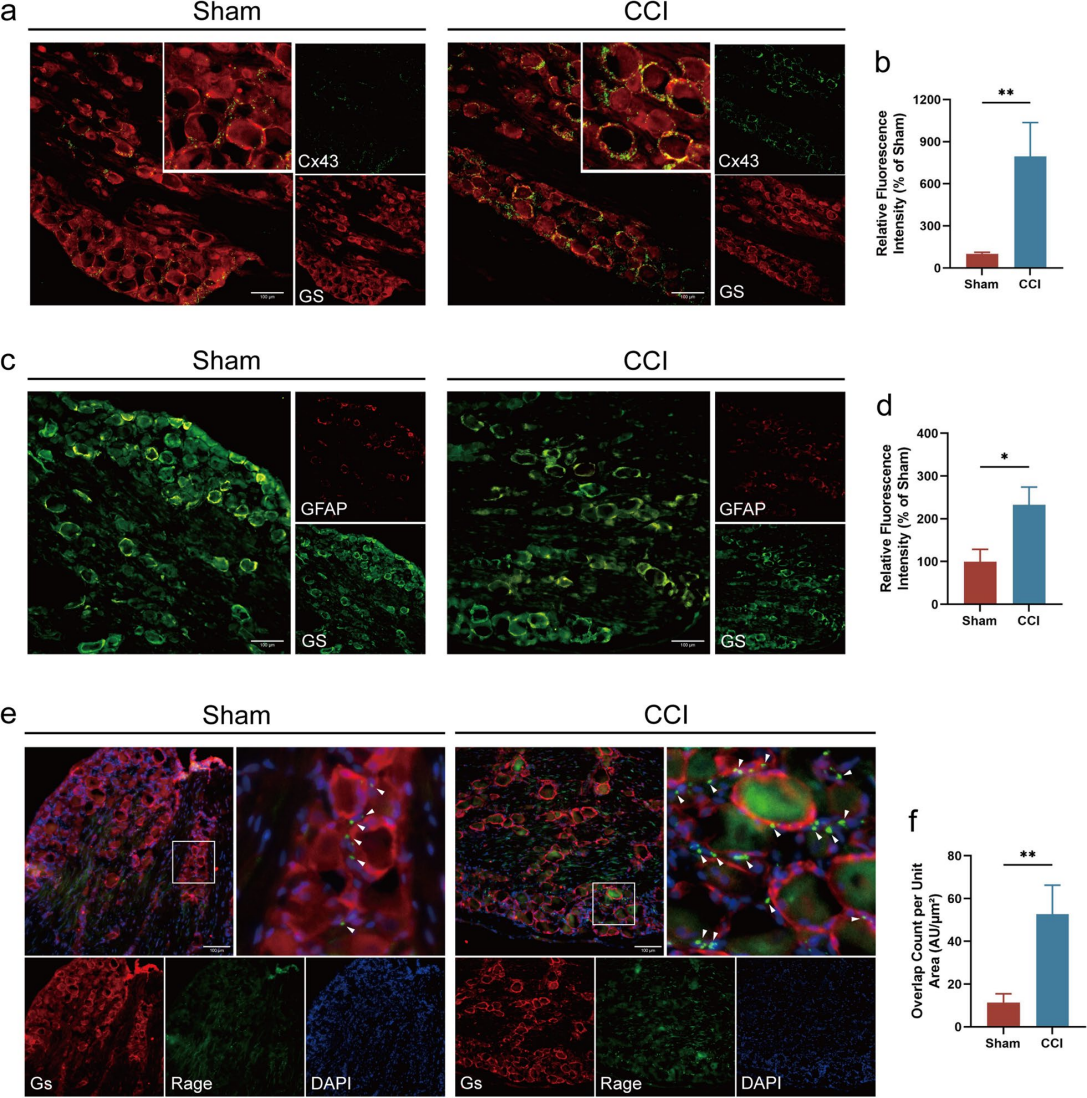
SGCs activation and upregulated RAGE expression in the DRG of CCI rats. **a** Immunofluorescence staining illuminated the expression of Cx43 and GS in the L2–L5 DRG of CCI and Sham rats (scale bar, 100 μm). **b** Fluorescence expression intensity of Cx43 was analyzed by ImageJ. **c** The expression of GFAP and GS in the L2–L5 DRG (scale bar, 100 μm). **d** Fluorescence expression intensity of GFAP was analyzed by ImageJ. **e** Immunofluorescence staining illuminated the expression of GS and RAGE in the L2–L5 DRG (scale bar, 100 μm). **f** Quantify the colocalization of RAGE and GS fluorescence per unit area analyzed by ImageJ. Cx43, connexin 43; GFAP, glial fibrillary acidic protein; RAGE, receptor for advanced glycation end products; GS, glutamine synthetase

SGCs, which surround DRG neuron cell bodies, play a crucial role in pain modulation (Qiu et al. 2024; Schmitt et al. 2020). Immunofluorescence staining revealed significantly elevated levels of Cx43 (Fig. 2a, b) and GFAP (Fig. 2c, d), markers of SGC activation, in the DRG of CCI rats. Additionally, RAGE, the receptor mediating biological effects of AOPPs, was predominantly observed in SGCs and was increased in the DRG of CCI rats compared to Sham rats (Fig. 2e, f). These results indicate a correlation between SGCs activation and RAGE expression in NP development following CCI.

### AOPPs induce SGCs activation in vitro

**Fig. 3:**
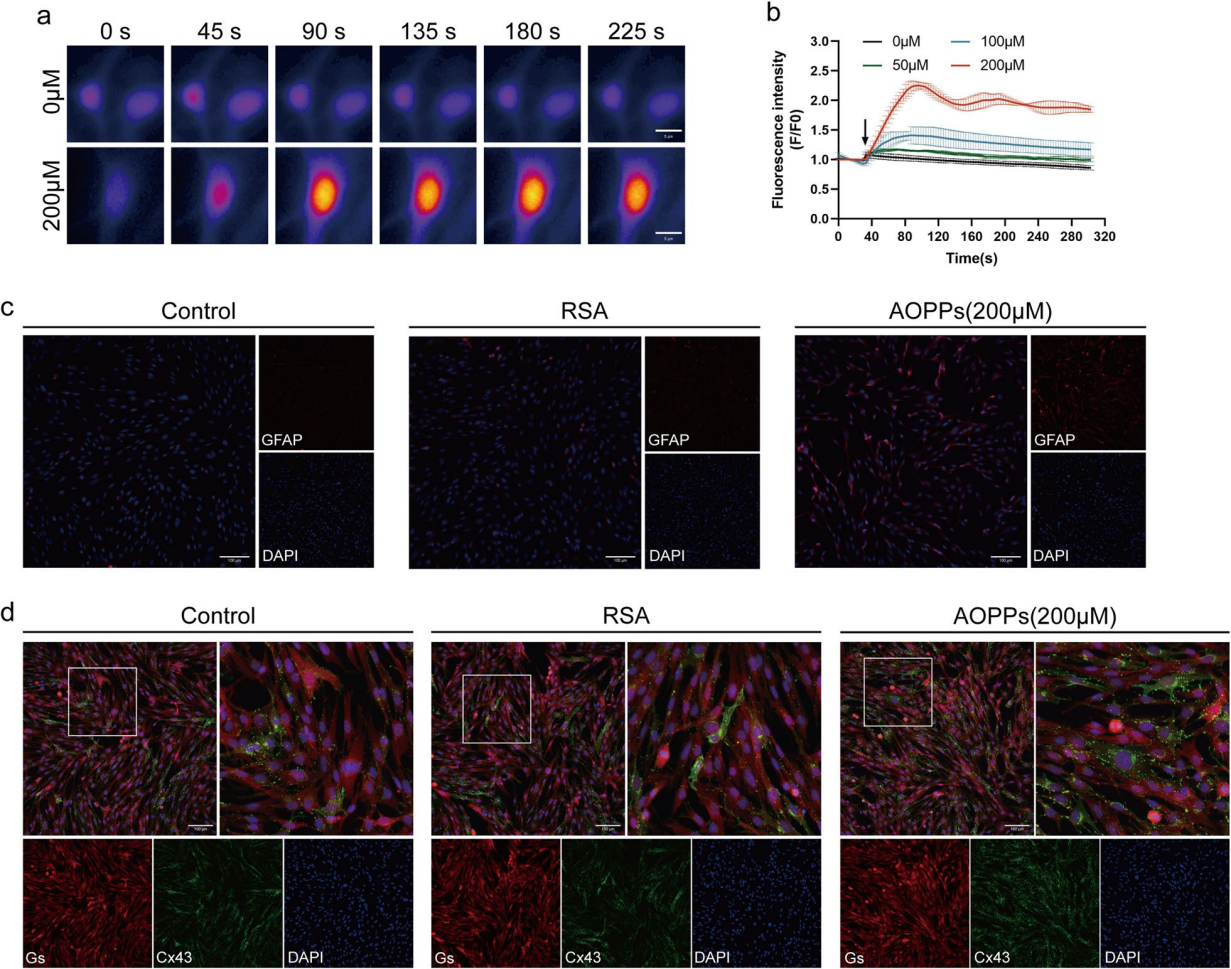
AOPPs induce SGCs activation in vitro. **a** Representative confocal images of Fluo4-AM fluorescence were recorded for 300 s in treated with AOPPs for 24 h and exposed to a pulse of 2 μM free Ca^2+^ (n = 6, Scale bar, 5 µm). **b** Fluorescence dynamics analyzed using ImageJ software from (**a**). **c** Immunofluorescence showing GFAP-positive cell after AOPPs treatment in SGCs (Scale bar, 100 µm). **d** Immunofluorescence illuminated the Cx43 expression after AOPPs treatment in SGCs (Scale bar, 100 µm). RSA, Rat serum albumin

To investigate the relationship between AOPPs accumulation and SGC activation, primary SGCs were cultured in *vitro*. As shown in Fig. 3a, b, AOPPs intervention promoted calcium influx in primary SGCs in a concentration-dependent manner. Immunofluorescence analysis revealed that AOPPs increased proportions of GFAP-positive SGCs (Fig. 3c) and upregulated Cx43 expression (Fig. 3d) in SGCs. These results indicate that AOPPs can directly induce SGCs activation in vitro.

### AOPPs induce ROS production and inflammatory cytokine release in SGCs

**Fig. 4:**
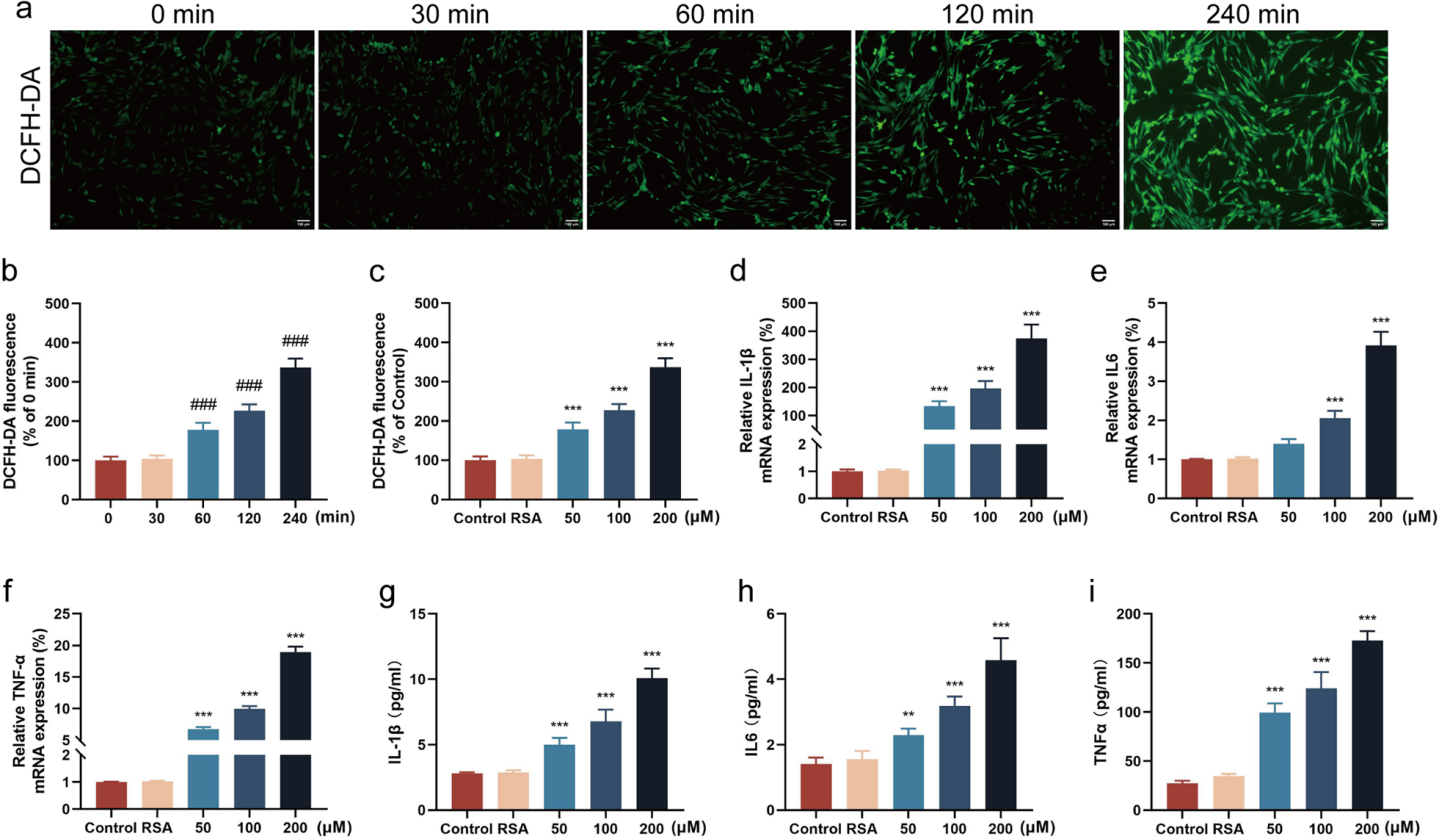
AOPPs induced ROS generation and inflammatory cytokine release in SGCs. **a** Representative confocal images of DCFH-DA fluorescence after AOPPs treatment (Scale bar, 100 µm). **b** Fluorescence intensity statistics following 200 μM AOPPs treatment for various durations. **c** Fluorescence intensity statistics after 240 min of treatment with different AOPPs concentrations. **d**, **f** qPCR analysis of IL-1β, IL-6, and TNF-α expression after treatment with different AOPPs concentrations (n = 3, one-way ANOVA). **g–i** ELISA analysis of IL-1β, IL-6, and TNF-α release following treatment with different AOPP concentrations (n = 6, one way-ANOVA). Data are presented as mean ± standard error of the mean (SEM). ###*p* < 0.01 vs 0 min group, **p < 0.01, ***p < 0.001 vs RSA group. IL-1β, interleukin 1β; IL-6, interleukin 6; TNFα, tumor necrosis factor α

Excessive ROS accumulation contributes to nerve injury and inflammatory responses (Wang et al. 2024). Thus, we assessed ROS production in SGCs following AOPPs exposure, and observed a time- and concentration-dependent increase in ROS levels (Fig. 4a–c). Furthermore, AOPPs treatment upregulated mRNA expression levels of IL-1β, IL-6, and TNF-α in SGCs (Fig. 4d, f), and induced release of these above inflammatory factors (Fig. 4g, h). These results suggest that AOPPs induce ROS generation and inflammatory cytokine release in SGCs, thereby contributing to the development of NP.

### AOPPs induce SGCs activation via RAGE/NF-κB signaling

**Fig. 5:**
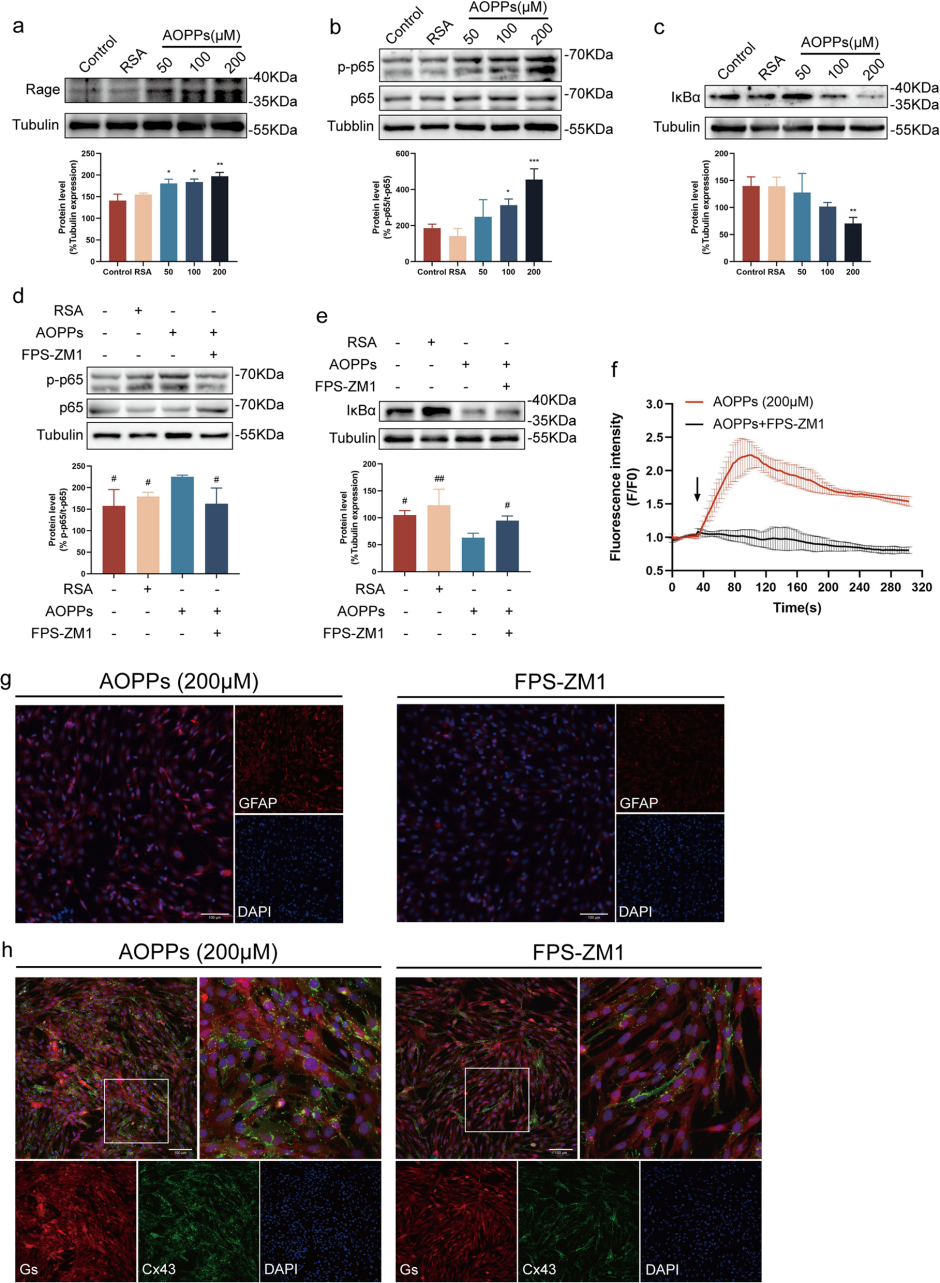
AOPPs activated SGCs through RAGE/NF-κB signaling. **a–c** Western blot analysis of RAGE, p65, p-p65, and IκBα expression after intervention with different AOPPs concentrations. **d**, **e** Western blot analysis of p65, p-p65, and IκBα expression after treatment with FPS-ZM1 and AOPPs (200 μM). **f** Fluo4-AM fluorescence dynamic was recorded for 300 s in treated with FPS-ZM1 and AOPPs (200 μM). **g** Immunofluorescence illuminated the GFAP-positive cells after AOPPs and FPS-ZM1 treatment in SGCs (Scale bar, 100 µm). **h** Immunofluorescence showing Cx43 expression by AOPPs and FPS-ZM1 treatment in SGCs (Scale bar, 100 µm). Data are presented as mean ± standard error of the mean (SEM). *p < 0.05, **p < 0.01 vs RSA group, #*p* < 0.05, ##*p* < 0.01 vs AOPPs group

RAGE has been implicated in NP development (Brederson et al. 2016). We investigated the impact of AOPPs on RAGE expression in SGCs, found that RAGE expression increased with AOPPs in a concentration-dependent manner (Fig. 5a). NF-κB constitutes a classical pro-inflammatory signaling pathway (Liu et al. 2017). AOPPs treatment induced phosphorylation of p65 (Fig. 5b) and downregulated IκBα expression (Fig. 5c) in a concentration-dependent manner, which were attenuated by a specific RAGE inhibitor, FPS-ZM1 (Fig. 5d, e). Furthermore, FPS-ZM1 treatment also mitigated AOPPs-induced calcium influx (Fig. 5f), decreased GFAP-positive SGCs proportions (Fig. 5g), and suppressed Cx43 expression (Fig. 5h). These findings suggest that AOPPs activate SGCs via the RAGE/NF-κB signaling pathway.

### Chronic AOPPs loading aggravated hyperalgesia and neuroinflammation induced by CCI

**Fig. 6:**
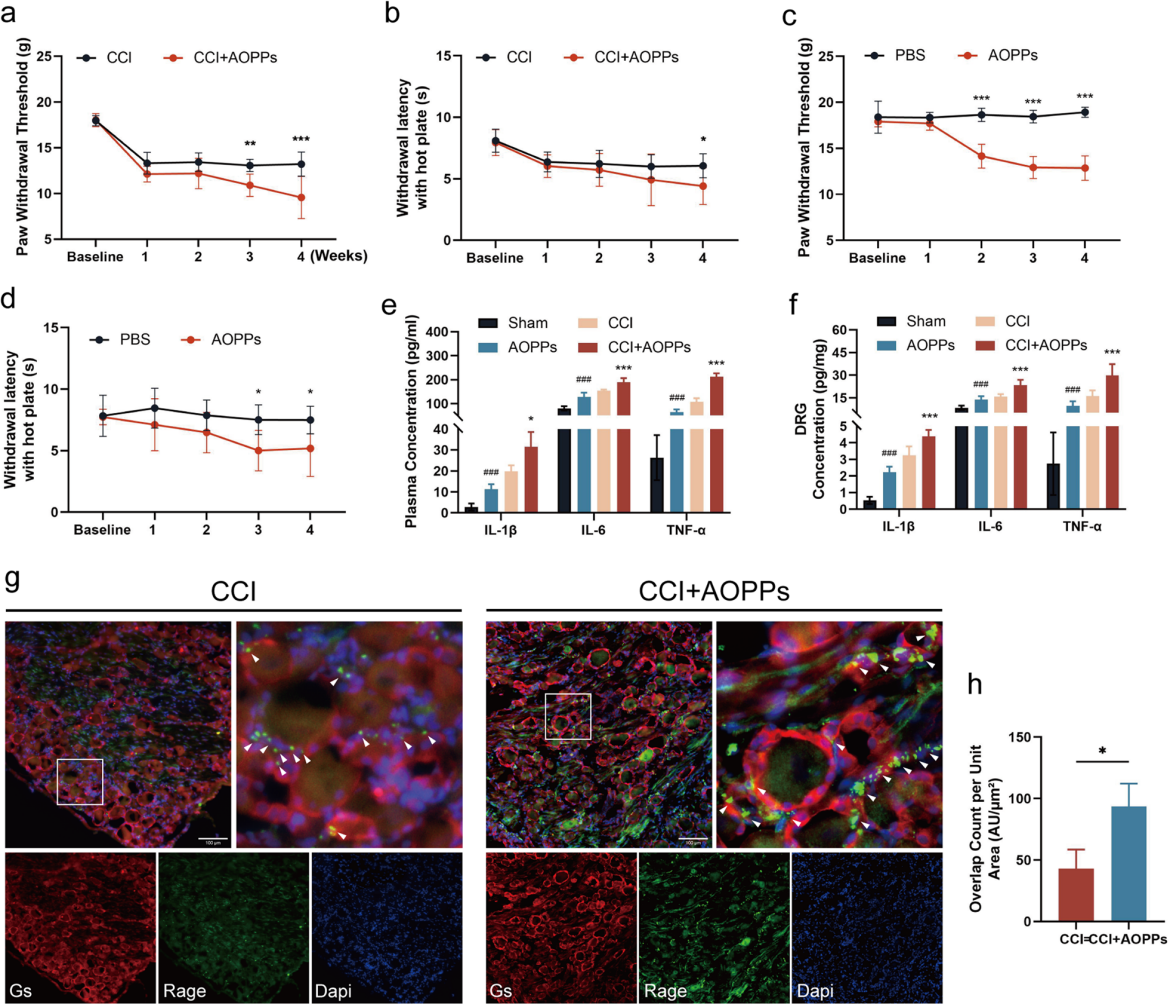
Chronic AOPPs loading aggravated hyperalgesia and neuroinflammation in CCI rats. **a** The mechanical withdrawal threshold (n = 8, two way-ANOVA), and **b** the thermal withdrawal latency (n = 8, two way-ANOVA) in CCI rats following 4 weeks of AOPPs loading. **c** The mechanical withdrawal threshold (n = 8, two way-ANOVA), and **d** the thermal withdrawal latency (n = 8, two way-ANOVA) in normal rats following 4 weeks of AOPPs loading. **e**, **f** ELISA analysis of IL-1β, IL-6, and TNF-α levels in the plasma and sciatic nerve of normal and CCI rats after AOPPs loading (n = 5, one way-ANOVA). **g** Immunofluorescence staining illuminated the expression of GS and RAGE in the L2–L5 DRG of CCI rats following AOPPs loading (scale bar, 100 μm). **f** Quantify the colocalization of RAGE and GS fluorescence per unit area analyzed by ImageJ. Data are presented as mean ± standard error of the mean (SEM). *p < 0.05, **p < 0.01, ***p < 0.001 vs CCI group or PBS group, ###*p* < 0.001 vs Sham group

Next, we observed the effects of AOPPs on NP induced by CCI. Chronic AOPPs loading further reduced the mechanical paw withdrawal threshold (Fig. 6a) and thermal paw withdrawal latency (Fig. 6b) in CCI rats. Importantly, AOPPs loading also reduced these thresholds in Sham rats (Fig. 6c, d). Chronic AOPPs loading increased IL-1β, IL-6, and TNF-α levels in the plasma and DRG of both CCI and Sham rats (Fig. 6e, f). Moreover, RAGE expression in SGCs was further elevated in the CCI + AOPPs group compared to the CCI-only group (Fig. 6g, h). These results indicate that AOPPs exacerbate NP and enhance neuroinflammation, highlighting the role of the RAGE/NF-κB signaling pathway in NP.

## Discussion

NP affects 7–8% of the general population and constitutes 20–25% of chronic pain (Gilron et al. 2015). Despite its prevalence, the precise mechanisms of NP remain elusive. SGCs in peripheral ganglia have emerged as critical regulators of NP, orchestrating the microenvironment around neurons (Lu et al. 2023). Our study elucidated the correlation between AOPPs accumulation and NP using a CCI rat model, demonstrating that AOPPs exacerbate hyperalgesia via SGC activation, which is mediated through the RAGE/NF-κB signaling pathway. Our research shed light on the role and mechanisms of AOPPs in NP, suggesting their potential as biomarkers for nerve injury.

AOPPs initially identified in IgA nephropathy, with their accumulation predict poor prognoses (Cao et al. 2014). To date, AOPPs have emerged as potential biomarkers of disease pathology, that promoting further investigation into the toxicological mechanisms of AOPPs. AOPPs induce scorch death and inflammation of endothelial cells by inducing trophoblast to secrete small extracellular vesicles (Chen et al. 2023). AOPPs accumulation is correlate with decreased bone mineral density in postmenopausal women, predicting osteoporosis severity (Wu et al. 2015). Elevated AOPPs levels are associated with increased TRPV1 and CGRP expression in DRG of rats receiving complete Freund's adjuvant foot pad injections (Ding et al. 2016). Recent research linked AOPPs to mechanical allodynia and cold hyperalgesia in a mouse model of multiple sclerosis (Rodrigues et al. 2024). Here, we show that AOPPs are elevated in CCI rats and AOPPs accumulation exacerbate hyperalgesia. Therefore, AOPPs may play an important role in NP.

RAGE modulates pain regulation within the central nervous system, influencing nitroglycerin-induced migraine and hyperalgesia by altering blood–brain barrier permeability (Jeong et al. 2022; Chen et al. 2022b). In microglial cell, RAGE activation exacerbates NP induced by paclitaxel (Moraes et al. 2024). Evidence supports RAGE expression in DRG and its involvement in peripheral nerve injury-induced NP, although specific DRG tissue RAGE expression sites are unidentified (Araldi et al. 2024; Bestall et al. 2018; Allette et al. 2014; Li et al. 2016). It has been demonstrated that RAGE serves as a pivotal signaling receptor for AOPPs (Xie et al. 2013). Here, we first identified RAGE expression in SGCs and AOPPs promote RAGE expression in SGCs. Additionally, the RAGE inhibitor FPS-ZM1 mitigates AOPPs-induced calcium influx in SGCs, highlighting the critical role of RAGE in AOPPs-induced SGCs activation.

The Inflammatory response contributes to the onset of chronic pain following nerve injury (Sommer et al. 2018; Ji et al. 2016). Activation of SGCs after nerve injury results in release of inflammatory cytokines, increasing neuronal excitability and initiating pain behavior. These cytokines also enhance intercellular signaling and calcium flux by opening Cx43 channels, further promoting SGCs activation (Qiu et al. 2024; Spray and Hanani 2019). In this study, AOPPs exacerbate neuroinflammation in CCI rats and promoted ROS production and SGCs activation in vitro. The NF-κB signaling pathway drives chronic inflammation, involving p65 phosphorylation and subsequent IκBα degradation (Taniguchi and Karin 2018; Zandi et al. 1997). This pathway directly regulates TNF-α and IL-6 production and modulates IL-1β transcription and maturation (Takeuchi and Akira 2010; Hiscott et al. 1993). Previous studies have showed AOPPs induce synovial cell inflammation via NF-κB signaling and activate microglia inflammasomes post-spinal cord injury (Zheng et al. 2013; Liu et al. 2020). Here, we present that AOPPs promotes p65 phosphorylation and IκBα degradation in SGCs, which are partially attenuated by FPS-ZM1 treatment, underscoring essential role of RAGE/NF-κB signaling in AOPPs-induced inflammation.

Summary, our research underscores the pivotal role of AOPPs in NP by activating SGCs and promoting inflammatory cytokine release through the RAGE/NF-κB signaling pathway. These finding contribute to understanding the mechanisms underlying the pathogenesis of NP. Reducing AOPPs accumulation and its cascading effects may be helpful for ameliorating NP.

## Data Availability

No datasets were generated or analysed during the current study.
